# Inclusive Intimacy: Sexual Experiences, Debut, and Partners Among Females Ages 15–25 with and Without Disability, NSFG 2011–2019

**DOI:** 10.1007/s11195-025-09931-9

**Published:** 2026-02-18

**Authors:** J. Dalton Stevens, Anne Valentine, Jessica N. Hoyle, Frank S. Li, Kelsey Zionskowski, John A. Harris, Ilhom Akobirshoev, Monika Mitra, Willi Horner-Johnson

**Affiliations:** 1 Department of Sociology, Anthropology, and Social Work, Texas Tech University, Lubbock, USA; 2 Lurie Institute for Disability Policy, Heller School for Social Policy and Management, Brandeis University, Waltham, USA; 3 Institute on Development and Disability, Oregon Health and Science University, Portland, USA; 4 Department of Obstetrics, Gynecology, and Reproductive Science, School of Medicine, Magee-Womens Research Institute, Pittsburgh, USA

**Keywords:** Disability, Sexual and reproductive health, Sexual experiences, Sexuality, Adolescence and early adulthood

## Abstract

This study extends the growing literature on adolescent and young adult females with disabilities’ sexual experiences by using nationally representative survey data. We analyzed the 2011–2019 National Survey of Family Growth to examine disability’s association with sexual experiences among females aged 15–25 (*n* = 7884). Chi-square and modified Poisson regression analysis compared sexual experiences, debut, and number of partners of females with and without disability by status and type. After adjustment, females with any, cognitive, or physical/sensory disability were more likely than those without disability to have had an opposite-sex sexual debut before age 15 and 10 or more opposite-sex sex partners. Those with any or cognitive disability were more likely than those without disability to have given oral sex to a male, receive oral sex from a male, had anal sex with a male, had any sexual experience with a female, and had 10 or more same-sex partners. Our analyses provide detailed population-level data on disabled adolescent and young adult females’ sexual experiences, debut, and partners and demonstrate that young disabled females have active sexual lives, highlighting the need for comprehensive sexual education and health care addressing relationships with all genders, STI testing and prevention, and sexual rights and consent.

**Supplementary Information:**

The online version contains supplementary material available at 10.1007/s11195-025-09931-9.

## Introduction

Becoming sexually active is a developmental process typically occurring in adolescence or young adulthood [1]. Disabled^1^ females^2^ face challenges to sexual expression, such as overmedicalization, bias against disabled people as sexual partners, increased risk of sexual abuse, and internalized ableism [2–6]. Widespread assumptions label disabled females as non-sexual, due to paternalistic views that infantilize disabled people [3, 7]. Ableist stereotypes limit the sexual rights of females with various types of disability by restricting access to accurate information and opportunities for healthy sexual relationships. Many disabled females receive limited sexual education prior to first intercourse [8]; lack privacy due to living arrangements [9]; and encounter attitudinal barriers from parents, guardians, care workers, teachers, and health care professionals [3, 5, 7]. These factors may impact sexual and reproductive health across disability status by decreasing knowledge and communication about sex and therefore constraining sexual and reproductive rights, sexual activity, and healthy sexual lives [4, 7, 8, 10].

Despite obstacles, disabled females lead active sexual lives, documented in growing interdisciplinary literature [3, 6, 8, 11–15]. Findings regarding patterns of sexual activity vary considerably across studies. Kahn and colleagues’ [12, 13] analysis of Add Health data showed that females with physical disabilities and cognitive disabilities had lower odds of having vaginal sex, oral sex, anal sex, or any sexual experience than their peers without disabilities while those with mild and moderate disability experienced similar rates as those without disability across sexual activities. Argenyi and colleagues [14] found little difference in sexual experiences between college students with and without disability. More recently, research using the Oregon Healthy Teens survey reported that students with disability were more likely than those without disability to identify as lesbian, gay, or bisexual and to have ever had sex, sex before the age of 15, two or more sexual partners total, two or more partners within the past 3 months, used drugs or alcohol at last intercourse, and sex without condoms [11].

The mixed findings underscore the need to better understand sexual experiences among adolescent and young adult females with disabilities to help craft tailored and robust sex education, improve reproductive and sexual health care practice, and develop strategies to enhance sexual rights while addressing risks. Where other studies have focused on individual disability groups, used data from a single state, or analyzed non-representative national samples [11–14], the purpose of this study is to use nationally representative survey data to compare the sexual experiences of adolescent and young adult females with and without any, cognitive, and physical and/or sensory disability. Previous literature suggests differing sexual experiences, debut, and number of partners based upon disability status, but differences have not been assessed with nationally representative data. Our results can inform various stakeholders involved in addressing the sexual and reproductive education and health care needs of this population.

## Methods

### National Survey of Family Growth

**Fig. 1:**
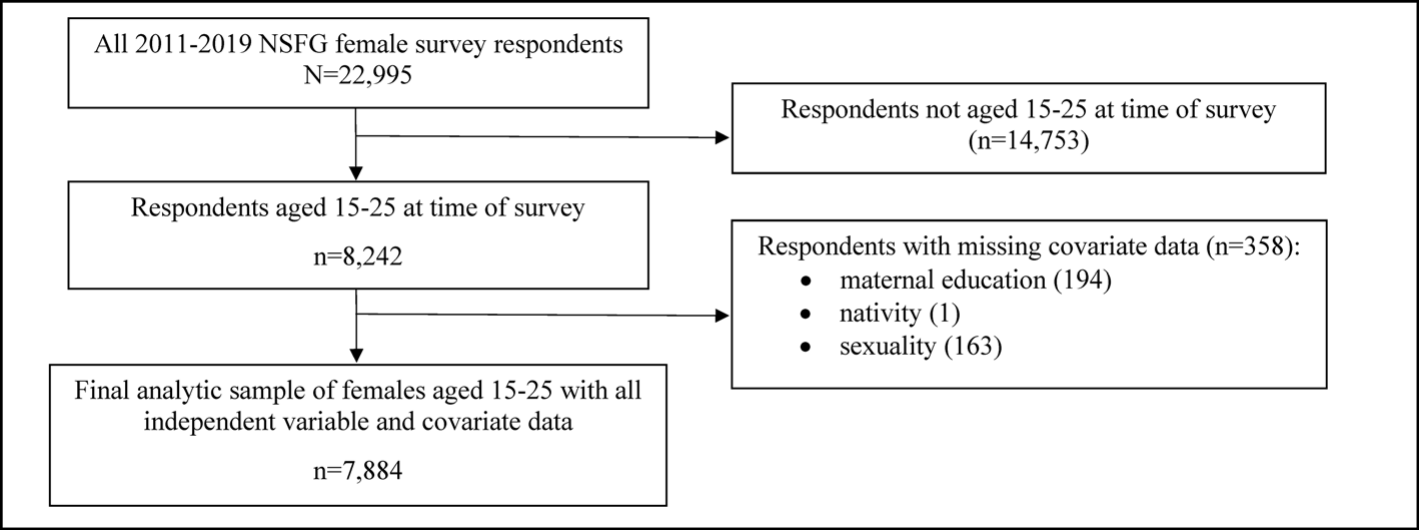
Analytic sample selection process, National Survey of Family Growth, 2011–2019

The National Survey of Family Growth (NSFG) is a nationally representative household survey implemented on a continuous basis since 2006 by the Centers for Disease Control and Prevention’s National Center for Health Statistics. The NSFG is a cross-sectional survey that collects data on pregnancies, births, family planning, relationships, health, and sexual experiences. The individual level data are collected through interviews and computer-aided personal interviewing for more sensitive questions, such as those on sexual experiences. Disability accommodations are provided when needed. We analyzed data from the 2011–2019 waves, the only waves including disability data that can be combined for accurate population estimates. The average response rate for these waves was 67.7% [16]. The 2011–2019 female respondent file included 22,995 observations. After restricting to adolescents and young adults aged 15–25 and excluding those with missing data on study variables, the total sample size for this study was 7,884 respondents. Figure 1 shows the process of arriving at the analytic sample. Because of the sensitive nature of the data, the sample size fluctuates by question and is indicated in Appendix A and main findings tables where it differs due to missing responses and exclusion of those with no sexual experience from being asked certain questions. This research was deemed exempt by the Brandeis University IRB under category 4—secondary data analysis.

### Measures

#### Disability

The NSFG uses six questions to assess disability status among respondents that align with a mobility disability, hearing disability, vision disability, cognitive disability, self-care disability, and independent living disability [17]. In line with previous literature [18], we created four analytic groups for this study: those without any disability, those with any disability, those with cognitive disability, and those with a physical and/or sensory disability (physical/sensory disability herein). We categorized respondents as having no disability if they answered no to all six questions and any disability if they answered yes to any of the six questions. The cognitive disability group included those responding yes to having difficulty concentrating, remembering, or making decisions. The physical/sensory disability group included those who responded yes to having difficulty seeing, even when wearing glasses or contacts; hearing; or walking or climbing stairs. We had to combine the physical and sensory disability categories due to low frequency within the data. The disability groups are not mutually exclusive.

#### Sexual Experiences

The NSFG includes questions focused on specific sexual experiences with opposite-sex and same-sex partners. These include any receptive vaginal sex with a male, giving oral sex to a male, receiving oral sex from a male, having anal sex with a male, having any sexual experience with a female, giving oral sex to a female, and receiving oral sex from a female. Responses were dichotomous (yes/no), indicating whether respondents had ever had these experiences or not. The NSFG asks about opposite-sex and same-sex sexual debut and number of partners. We coded opposite-sex and same-sex sexual debut as 14-years-old or younger or 15-years-old or older. In the 2017–2019 data wave, the number of same-sex partners was top coded to 10 or more. To harmonize data across waves and partner genders and to reflect the number of lifetime sexual partners associated with higher odds of cancer, sexually transmitted infections, and related diagnoses [19], we used a binary variable for number of sexual partners, coded as < 10 or ≥ 10 or more opposite-sex or same-sex sex partners. While the base analytic sample is 7,884, sample sizes differ across these measures due to refusals, “don’t know” responses, and inapplicability based on other responses (e.g., respondents were not asked how many partners they had if they report never having had receptive vaginal intercourse).

#### Demographic and Socioeconomic Covariates

Demographic variables include a measure for age group, poverty status, maternal education, race and/or ethnicity (race/ethnicity herein), nativity, and sexual orientation. We grouped age into three categories: 15–17-years old, 18–21-years old, and 22–25-years old. For family poverty status, respondents are in one of four groups: < 99% of the Federal Poverty Level (FPL), 100–199% of the FPL, 200–399% of the FPL, or ≥ 400% of the FPL. We grouped maternal education into four groups: less than high school, high school graduate, some college, and college graduate. Race/ethnicity includes groups for non-Hispanic (NH) White, NH Black, Hispanic, and NH other/multi-racial [8]. We dichotomized nativity into two groups: (1) US born or (2) immigrant. For sexual orientation, we use the “orient” variable for waves 2011–2017 and “orient_a” and “orient_b” for the 2017–2019 data wave to create groups for people self-identified as heterosexual, lesbian, or bisexual. We excluded respondents from the analysis who refused to answer, answered that they did not know, or identified as “something else” due to low frequency and uncertainty of their orientation. Without a gender identity variable, we stick to the language “female” throughout the manuscript as the variables reflects sex.

### Analyses

We used chi-square tests to compare demographic, socioeconomic, and sexual experience characteristics of adolescent and young adult females with and without disabilities. We also stratified by disability type, comparing those with cognitive disability and physical/sensory disability to those without disability. We applied NSFG survey weights for all analyses to provide national estimates. We used modified Poisson regression analysis to assess the comparative likelihood of sexual experiences among those with any, cognitive, and physical/sensory disability compared to those without disability [20]. We estimated the unadjusted and adjusted prevalence ratios (aPR) and 95% confidence intervals (CIs) for all outcome variables. Covariates included in multivariable models for adjusted estimates included age group, poverty status, maternal education, race/ethnicity, nativity, sexuality, and data wave. We estimated all models with robust standard errors. All analyses were conducted in STATA 18.0 MP (College Station, TX).

## Results

The analytic sample consisted of 7,884 total respondents, including 1,809 (21.5%) with any disability, 1,314 (16.1%) with cognitive disability, and 695 (8.2%) with physical/sensory disability. Table 1 shows the weighted demographic and socioeconomic characteristics for the sample. Females with cognitive disability tended to be slightly younger than those without disability. Respondents with any, cognitive, or physical/sensory disability had lower maternal education than those without disability, and were more likely than those without disability to live below the FPL. Those with any, cognitive, or physical/sensory disability were much more likely to identify as lesbian or bisexual than those without disability.

**Table 1:** Weighted sample characteristics among females with and without any disability, cognitive disability, and physical/sensory disability, NSFG 2011-2019 (n = 7884)

Characteristic	Total sample (*N* = 7884)		No disability (*n* = 6075)		Any disability (*n* = 1809)		Cognitive disability (*n* = 1314)		Physical/sensory disability (*n* = 695)	
	*N*	Weighted %	*N*	Weighted %	*N*	Weighted %	*N*	Weighted %	*N*	Weighted %
*Age group*										
15–17 years old	2218	(25.0%)	1644	(24.3%)	574	(27.5%)	450	(30.2%)	187	(21.3%)
18–21 years old	2733	(36.7%)	2083	(36.1%)	650	(38.7%)	462	(38.8%)	241	(39.0%)
22–25 years old	2933	(38.4%)	2348	(39.6%)	585	(33.8%)	402	(31.1%)	267	(39.7%)
						*p = 0.011		***p = 0.000		*p* = 0.311
*Maternal education*										
Less than high school	1554	(16.4%)	1172	(15.9%)	382	(18.4%)	273	(18.2%)	160	(21.2%)
High school graduate	2284	(27.3%)	1734	(26.7%)	550	(29.4%)	405	(29.8%)	216	(28.7%)
Some college	2148	(27.9%)	1636	(27.7%)	512	(28.5%)	372	(29.3%)	191	(28.9%)
College graduate	1898	(28.3%)	1533	(29.6%)	365	(23.6%)	264	(22.8%)	128	(21.1%)
						**p = 0.008		*p = 0.019		*p = 0.016
*Family poverty status*										
99% FPL or lower	2934	(32.6%)	2133	(30.6%)	801	(40.0%)	594	(39.9%)	323	(41.1%)
100%–199% FPL	1989	(24.9%)	1542	(24.6%)	447	(25.6%)	326	(26.3%)	161	(22.7%)
200%–399% FPL	1878	(26.6%)	1524	(27.6%)	354	(23.0%)	250	(23.5%)	135	(24.3%)
400% FPL or higher	1083	(15.9%)	876	(17.1%)	207	(11.4%)	144	(10.3%)	76	(11.9%)
						***p = 0.000		***p = 0.000		*p = 0.011
*Race-ethnicity*										
Non-hispanic white	3335	(52.8%)	2545	(52.8%)	790	(52.7%)	590	(52.9%)	256	(50.0%)
Non-hispanic black	1633	(14.4%)	1273	(14.7%)	360	(13.3%)	241	(11.8%)	174	(17.2%)
Hispanic	2190	(22.5%)	1704	(22.2%)	486	(23.5%)	349	(23.6%)	193	(23.7%)
Non-hispanic other	726	(10.4%)	553	(10.3%)	173	(10.5%)	134	(11.8%)	72	(9.1%)
						*p* = 0.688		*p* = 0.146		*p* = 0.302
*Nativity*										
US Born	7119	(90.8%)	5443	(90.5%)	1676	(92.2%)	1224	(92.1%)	634	(91.2%)
Immigrant	765	(9.2%)	632	(9.5%)	133	(7.7%)	90	(7.9%)	61	(8.8%)
						*p* = 0.154		*p* = 0.266		*p* = 0.838
*Sexuality*										
Straight	6736	(86.1%)	5370	(88.9%)	1366	(75.6%)	959	(73.0%)	548	(77.4%)
Lesbian	206	(2.5%)	127	(1.9%)	79	(4.8%)	66	(5.1%)	33	(5.02%)
Bisexual	942	(11.4%)	578	(9.2%)	364	(19.7%)	289	(21.9%)	114	(17.6%)
						****p = 0.000		***p = 0.000		***p = 0.000
*Data wave*										
2011–2013	2123	(25.9%)	1687	(26.8%)	436	(22.9%)	305	(22.5%)	167	(21.9%)
2013–2015	2127	(26.4%)	1638	(26.9%)	489	(24.8%)	368	(25.4%)	186	(22.3%)
2015–2017	1776	(23.7%)	1352	(23.1%)	424	(26.2%)	309	(26.0%)	162	(27.2%)
2017–2019	1858	(23.9%)	1398	(23.3%)	460	(26.1%)	332	(26.1%)	180	(28.6%)
						*p = 0.047		*p* = 0.160		*p = 0.035

*p < 0.05, **p < 0.01, ***p < 0.001

**Table 2:** Weighted sexual experience outcomes among females ages 15–25 with and without any disability, cognitive disability, and physical/sensory disability, NSFG 2011–2019 (*n* = 7884)

Experiences	Total (*n* = 7884)		No disability (*n* = 6075)		Any disability (*n* = 1809)		Cognitive disability (*n* = 1314)		Physical/sensory disability (*n* = 695)	
	*N*	Weighted %	*N*	Weighted %	*N*	Weighted %	*N*	Weighted %	*N*	Weighted %
*Opposite-sex sexual experiences*										
Vaginal Intercourse										
No	2647	(32.6%)	2063	(33.2%)	584	(30.2%)	442	(32.0%)	204	(26.7%)
Yes	5237	(67.4%)	4012	(66.8%)	1225	(69.8%)	872	(68.0%)	491	(73.3%)
						*p* = 0.098		*p* = 0.755		*p = 0.024
Give oral sex										
No	3248	(37.9%)	2527	(38.6%)	721	(35.4%)	526	(36.1%)	288	(34.7%)
Yes	5237	(62.1%)	3520	(61.4%)	1079	(64.6%)	780	(63.9%)	405	(65.3%)
						*p* = 0.114		*p* = 0.361		*p* = 0.238
Receive oral sex										
No	3067	(37.0%)	2393	(37.7%)	683	(34.6%)	508	(36.2%)	262	(32.4%)
Yes	4771	(63.0%)	3520	(62.3%)	1117	(65.4%)	798	(63.8%)	431	(67.6%)
						*p* = 0.139		*p* = 0.658		*p* = 0.090
Anal sex										
No	6126	(77.1%)	4785	(78.1%)	1341	(73.4%)	956	(71.8%)	522	(76.4%)
Yes	1,726	(22.9%)	1269	(21.9%)	457	(26.6%)	348	(28.2%)	171	(23.6%)
						*p = 0.008		**p = 0.002		*p* = 0.770
10 or more sexual partners										
No	4840	(85.6%)	3775	(88.0%)	1065	(77.7%)	752	(76.5%)	422	(78.4%)
Yes	824	(14.4%)	547	(12.0%)	277	(22.3%)	214	(23.5%)	103	(21.5%)
						***p = 0.000		***p = 0.000		**p = 0.002
Sexual debut										
14 years old or younger	955	(16.2%)	627	(13.7%)	328	(25.0%)	243	(25.1%)	143	(30.5%)
15–17 years old	2945	(55.9%)	2286	(56.0%)	659	(55.8%)	478	(58.8%)	246	(47.7%)
18 years old or older	1337	(27.8%)	1099	(30.3%)	238	(19.2%)	151	(16.1%)	102	(21.7%)
						***p = 0.000		***p = 0.000		***p = 0.000
*Same-sex sexual experiences*										
Any sexual experience with same-sex partner										
No	6394	(82.1%)	5078	(84.5%)	1316	(73.4%)	928	(71.6%)	521	(74.3%)
Yes	1477	(17.9%)	986	(15.5%)	491	(26.6%)	384	(28.4%)	174	(25.7%)
						***p = 0.000		***p = 0.000		***p = 0.000
Give oral sex										
No	6977	(89.3%)	5464	(90.6%)	1513	(84.8%)	1083	(84.1%)	576	(82.6%)
Yes	894	(10.7%)	600	(9.4%)	294	(15.2%)	229	(15.9%)	119	(17.4%)
						***p = 0.000		***p = 0.000		***p = 0.000
Receive oral sex										
No	6827	(88.0%)	5364	(89.5%)	1463	(82.7%)	1052	(82.2%)	564	(83.0%)
Yes	1047	(12.0%)	703	(10.5%)	344	(17.3%)	260	(17.8%)	131	(17.0%)
						***p = 0.001		***p = 0.000		**p = 0.009
10 or more sexual partners										
No	1414	(97.1%)	952	(97.9%)	462	(95.3%)	360	(94.5%)	164	(96.3%)
Yes	43	(2.9%)	22	(2.1%)	21	(4.7%)	18	(5.5%)	7	(3.7%)
						*p = 0.031		**p = 0.008		*p* = 0.552
Sexual debut										
14 years old or younger	355	(24.3%)	230	(22.8%)	125	(27.6%)	96	(27.8%)	52	(34.8%)
15–19 years old	809	(54.7%)	518	(52.3%)	291	(59.7%)	230	(59.2%)	99	(54.7%)
20 years old or older	291	(20.9%)	225	(24.8%)	66	(12.7%)	51	(12.9%)	21	(10.5%)
						**p = 0.001		*p = 0.017		*p = 0.019

*p < 0.05, **p < 0.01, ***p < 0.001

Chi-square tests (Table 2) indicated that females with physical/sensory disability (73.3%) were more likely than those without disability (66.8%) to have had vaginal intercourse with a male. Those with disability were statistically no more or less likely than those without disability to give or receive oral sex to males. Respondents with any disability (26.6%) and cognitive disability (28.2%) were more likely than those without disability (21.9%) to report having previous anal sex encounters with a male. Those with any disability (22.3%), cognitive disability (23.5%), or physical/sensory disability (21.5%) were more likely than those without disability (12.0%) to have had 10 or more male sexual partners. Similarly, respondents with any disability (25.0%), cognitive disability (25.1%), and physical/sensory disability (30.5%) were more likely than those without disability (13.7%) to have had heterosexual sexual debut at age 14-years-old or earlier. Those with any disability, cognitive disability, or physical/sensory disability were slightly more likely than those without disability to have any same-sex sexual experience, give oral sex to another female, receive oral sex from another female, and have ten or more sexual partners of the same-sex. While those with any (27.6%), cognitive (27.8%), or physical/sensory (34.8%) disability were slightly more likely than those without disability (22.8%) to have sexual debuts with other females at age 14 or younger.

Table 3 presents both unadjusted and adjusted prevalence ratios (aPRs) for sexual experiences among respondents with any disability, cognitive disability, or physical/sensory disability compared to those without disability.

**Table 3:** Weighted prevalence ratios from modified Poisson regression analysis for sexual experience among females ages 15–25 with and without disability by Type, NSFG 2011–2019 (*N* = 7,884)

SRH outcomes	Any Disability (*n* = 1809)		Cognitive disability (*n* = 1314)		Physical/Sensory Disability (*n* = 695)	
	Unadjusted PR (95% CI)	Adjusted PR (95% CI)	Unadjusted PR (95% CI)	Adjusted PR (95% CI)	Unadjusted PR (95% CI)	Adjusted PR (95% CI)
*Opposite-sex sexual experiences*						
Vaginal intercourse	(*n* = 7884)^a^		(*n* = 7884)		(*n* = 7884)	
	1.04 (0.99–1.10)	**1.09** **(1.04–1.14)**	1.01 (0.95–1.07)	**1.08** **(1.03–1.14)**	**1.10** **(1.02–1.17)**	1.06 (1.00-1.13)
Give oral sex	(*n* = 7847)		(*n* = 7847)		(*n* = 7847)	
	1.05 (1.00-1.11)	**1.10** **(1.05–1.16)**	1.03 (0.97–1.10)	**1.11** **(1.05–1.18)**	1.06 (0.97–1.15)	1.04 (0.96–1.13)
Receive oral sex	(*n* = 7847)		(*n* = 7847)		(*n* = 7847)	
	1.05 (0.99–1.11)	**1.10** **(1.04–1.15)**	1.02 (0.95–1.08)	**1.09** **(1.02–1.16)**	**1.08** **(1.00-1.17)**	1.07 (0.99–1.14)
Anal sex	(*n* = 7852)		(*n* = 7852)		(*n* = 7852)	
	**1.22** **(1.07–1.40)**	**1.22** **(1.07–1.39)**	**1.29** **(1.12–1.50)**	**1.33** **(1.15–1.53)**	1.03 (0.84–1.27)	0.97 (0.79–1.19)
Sexual debut before age 15	(*n* = 5237)		(*n* = 5237)		(*n* = 5237)	
	**1.82** **(1.52–2.19)**	**1.55** **(1.29–1.86)**	**1.72** **(1.41–2.11)**	**1.41** **(1.15–1.74)**	**2.06** **(1.63–2.60)**	**1.83** **(1.45–2.32)**
10 or more sexual partners	(*n* = 5664)		(*n* = 5664)		(*n* = 5664)	
	**1.86** **(1.53–2.24)**	**1.88** **(1.56–2.25)**	**1.88** **(1.53–2.30)**	**1.93** **(1.58–2.35)**	**1.58** **(1.20–2.06)**	**1.54** **(1.19–1.99)**
*Same-sex sexual experiences*						
Any sexual experience with same sex partner	(*n* = 7871)		(*n* = 7871)		(*n* = 7871)	
	**1.71** **(1.49–1.97)**	**1.23** **(1.09–1.38)**	**1.79** **(1.54–2.08)**	**1.25** **(1.09–1.42)**	**1.50** **(1.22–1.84)**	1.12 (0.94–1.33)
Give oral sex	(*n* = 7871)		(*n* = 7871)		(*n* = 7871)	
	**1.71** **(1.49–1.97)**	1.02 (0.88–1.19)	**1.65** **(1.35–2.08)**	1.01 (0.85–1.20)	**1.73** **(1.34–2.23)**	1.16 (0.96–1.42)
Receive oral sex	(*n* = 7874)		(*n* = 7874)		(*n* = 7874)	
	**1.64** **(1.38–1.95)**	1.10 (0.94–1.19)	**1.64** **(1.35–1.98)**	1.07 (0.90–1.27)	**1.47** **(1.15–1.89)**	1.02 (0.83–1.26)
Sexual debut before age 15	(*n* = 1455)		(*n* = 1455)		(*n* = 1455)	
	1.21 (0.96–1.61)	1.06 (0.94–1.29)	1.20 (0.88–1.64)	1.05 (0.77–1.43)	**1.52** **(1.04–2.18)**	1.35 (0.93–1.94)
10 or more sexual partners	(*n* = 1457)		(*n* = 1457)		(*n* = 1457)	
	**2.30** **(1.12–4.73)**	**2.26** **(1.19–4.27)**	**2.74** **(1.32–5.67)**	**3.24** **(1.73–6.10)**	1.31 (0.52–3.32)	0.92 (0.34–2.52)

^a^ Sample size fluctuates based on the question asked. Due to the sensitive nature of sexual experience questions, respondents may have skipped questions leading to differences across outcome variable. Missing data includes 37 observations from giving opposite-sex oral, 37 observations from receiving opposite-sex oral, 32 observations from opposite-sex anal, 2,647 observations from opposite-sex sexual debut, 2,220 observations from opposite-sex 10 or more sexual partners, 13 observations from any same-sex sexual experience, 13 observations from giving same-sex oral, 10 observations from receiving same-sex oral, 6,427 observations from same-sex sexual debut, and 6,429 observations from same-sex 10 or more sexual partners. See Appendix A for details on why data were missing (e.g., those who were not sexually active were not asked questions about age of debut or number of sexual partners)

Bold indicates significance *p* < 0.05 or lower

Adjusted for age group, poverty status, maternal education, race/ethnicity, nativity, sexuality, and data wave

We found some similarities and differences across disability status and type among adolescent and young adult females regarding opposite-sex sexual experiences. Respondents with any disability (aPR 1.09; 95% CI 1.04–1.14; *p* < 0.001) or cognitive disability (aPR 1.08; 95% CI 1.03–1.14; *p* < 0.001) were slightly more likely than those without disability to experience vaginal intercourse with a male while those with physical/sensory disability were no more or less likely than those without disability. Those with any disability (aPR 1.10; 95% CI 1.05–1.16; *p* < 0.001) and cognitive disability (aPR 1.11; 95% CI 1.05–1.18; *p* < 0.001) were more likely than those without disability to have had given oral sex to a male. Those with any (aPR 1.10; 95% CI 1.04–1.15; *p* < 0.001) disability were more likely than those without disability to receive oral sex from a male, as were those with cognitive (aPR 1.09; 95% CI 1.02–1.16; *p* < 0.01) disability specifically. Those with any disability (aPR 1.22; 95% CI 1.07–1.40; *p* < 0.01) and cognitive disability (aPR 1.33; 95% CI 1.15–1.53 *p* < 0.001) were more likely than those without disability to have past experiences of anal sex with a male. Regardless of disability type, those with disability were significantly more likely to have opposite-sex sexual debut before age 15 than those without disability, with those with cognitive disability having the highest adjusted prevalence ratio (aPR 1.93; 95% CI 1.58–2.35; *p* < 0.001). Similarly, all disability groups were more likely than those without disability to report having 10 or more male sex partners, but those with cognitive disability (aPR 1.93; 95% CI 1.58–2.30; *p* < 0.001) saw the highest difference compared to those without disability.

In terms of same-sex experiences, we observed significant variation across experiences by disability status and type. Those with any (aPR 1.23; 95% CI 1.09–1.38; *p* < 0.001) or cognitive (aPR 1.25; 95% CI 1.09–1.42; *p* < 0.001) disability were more likely than those without disability to have any same-sex sexual experience, but those with physical/sensory disability (aPR 1.12; 95% CI 0.94–1.33; *p* > 0.05) were no more or less likely than those without disabilities to have any same-sex sexual experience. Across disability type, disabled respondents were no more or less likely to give or receive oral sex to same-sex partners than those without disabilities in adjusted models. Those with disability, across type, were no more or less likely than those without disability to have same-sex sexual debuts before age 15. Respondents with any disability (aPR 2.26; 95% CI 1.19–4.73; *p* < 0.05) or cognitive disability (aPR 3.24; 95% CI 1.73–6.10; *p* < 0.001) were significantly more likely than those without disabilities of having had ten or more female sexual partners, with those with cognitive disability seeing over a three-fold higher prevalence compared to those without disability.

## Discussion

Our results demonstrate the shared and varied sexual experiences of females with disabilities throughout adolescence and early adulthood compared to nondisabled females. The analysis helps address a longstanding ableist stereotype that suggests that people with disability are non-sexual [3, 7]. Our results suggest some variation among the sexual experiences of females with and without disabilities throughout adolescence and early adulthood. These findings have implications for sexual and reproductive health, education, and rights for females with disability.

Our study helps confirm and clarify parts of the literature on the sexual experiences of adolescent and young adult females with disability. Like other studies [11, 21], we found that those with disabilities had much higher prevalence of identifying as lesbian or bisexual than their counterparts without disability, and we showed that adolescent and young adult females with any or cognitive disability are comparatively more likely than those without disability to have any previous same-sex sexual encounter. Our findings also extend knowledge on the sexual risk taking of young people with disability; across disability types, disabled respondents were more likely than their non-disabled peers to debut before age 15 and have 10 or more opposite-sex sexual partners. Further, those with any or cognitive disability were more likely than respondents without disability to engage in anal sex, oral sex with males, and have 10 or more same-sex sexual partners. These findings are consistent with Horner-Johnson and colleagues [11] who found that disabled youth reported earlier sexual debut and higher number of partners than their peers without disability. We also find consistency with previous work that showed that college students with specific disabilities such as attention-deficit hyperactivity disorder and psychiatric conditions—likely to be represented in our cognitive disability group—were at higher risk than other students without disability of engaging in riskier sexual behavior [14].

Respondents across disability type had higher likelihood of having opposite-sex sexual debut younger than 15, highlighting the potential risk for coercive sexual experiences. Previous research shows that sexual debut at age 14 or younger is associated with higher rates of sexual assault and coercion, poor mental health outcomes, unsafe sexual practices, sexually transmitted infections (STIs), and childbearing in the teenage years [11, 22, 23]. Effective sex education, personal and family practices, healthy peer and partner communication, and improving community knowledge and safety may help address risks associated with early sexual debut among people with and without disability [24]. Tailored early comprehensive sexual education that includes instruction in healthy and respectful relationships for people with and without disability may help strengthen sexual decision-making, communication, and empower young people to report abuse [15, 25]. Integrating consent and disability-related information in general sexual education may help prevent coercive sexual practices that harm disabled females. Families are instrumental to developing children’s sexual and reproductive health, and having early and frequent conversations regarding sex, safety, consent, and healthy relationships can help provide a strong foundation [26].

Families, schools, clinical settings, and other organizations are positioned to assist in early screening strategies for sexual abuse [26, 27]. However, screening for sexual abuse is complicated, especially among younger populations. There are not only different types of screenings but contextual factors at play, such as where screenings should take place and who should be present [27, 28]. Research on and development of effective strategies to screen for sexual abuse among people with various disabilities are of great importance to promote the sexual and reproductive rights and safety of people with disabilities.

Considering the slightly higher prevalence of oral and anal sex among those with disability compared to those without, it is important to note various risk factors associated with specific sexual experiences. Many people who engage in oral or anal sex do not use condoms, exposing them to risk for STIs [29]. Oral sex can transmit human papillomavirus (HPV), herpes, hepatitis B, gonorrhea, syphilis, chlamydia, and HIV [30]. Research shows strong associations between infections, like HPV, and oropharyngeal cancer [31]. Using non-lubricated latex or plastic condoms or dental dams during oral sex can reduce the likelihood of transmission by providing a barrier that prevents direct contact, and products should remain available, accessible, and affordable [30]. Screening for oral STIs is important as few people use condoms during oral sex [29, 30]. Anal sex prevalence has grown in recent years, and our analyses suggest that adolescent and young adult females with any or cognitive disability engage in anal sex more than those without disability. STI risks are similar between anal and oral sex, and anal sex is associated with cancer risk in females [32, 33]. Additionally, research has found associations between heterosexual anal intercourse and risky sexual behaviors such as having multiple concurrent partnerships, not using a condom at last sex, and using less effective contraception methods [29, 34]. Frank and accessible discussion of safe oral and anal sex practices may help protect against risks.

Some same-sex sexual experiences were more prevalent among those with any disability or cognitive disability when compared to peers without disability. Consistent with previous research [11, 35], we found that respondents with disabilities were much more likely than nondisabled peers to identify as lesbian or bisexual. Those who are disabled and engage in sex with same-sex partners belong to two intersecting sociodemographic groups facing health disparities. Intersecting systems of oppression may lead to unique challenges related to accessing sexual and reproductive health information, utilizing sexual and general health services, and increased discrimination and stress [36, 37]. Because same-sex sexuality appears to be prevalent throughout adolescence and early adulthood for disabled people, it is important to integrate LGBTQ + information in sexual education to improve sexual decision-making, knowledge, and sexual health development. Having disability organizations and disability-led peer networks develop and disseminate LGBTQ + inclusive sexual education for people with various disabilities is far from commonplace and varies greatly among states [38]. As of 2023, only 10 states have policies to include sexual orientation affirming instruction on LGBTQ + identities or discussion on LGBTQ + sexual health [38]. However, providing LGBTQ + affirming instruction with input from disabled people is considered best practice to help young people learn about sex, disability, and identity and may reduce internal or external stigma around these identities [39, 40].

Our analysis demonstrates some differences in the number of opposite-sex and same-sex sexual partners of disabled respondents and nondisabled peers. Those with any, cognitive, or physical/sensory disability had comparatively higher rates of having 10 or more opposite-sex sexual partners than their peers without disability, and the same is true for 10 or more same-sex sexual partners for those with any or cognitive disability. Depending on the activity, having 10 or more sexual partners is associated with higher risk for chronic and/or life threatening illnesses like cancer [19]. Focusing education on using protection with each new partner during every encounter is of great importance and can help prevent STIs and unintended pregnancy [8, 25]. Because disabled people are more likely to have a high number of sexual partners, providing knowledge and tools to protect one’s self are important in protecting against STIs and downstream physical and mental health effects, such as HPV-related cancers and detrimental effects on one’s sense of self [31, 41].

### Limitations and Strengths

Our findings should be interpreted in the context of certain data limitations. First, all variables, including disability, were based on self-reported data. Disability was assessed through a series of six questions on functional limitations. These questions do not allow separate identification of specific categories such as mental health disability, intellectual disability, or diagnoses like attention-deficit hyperactivity disorder that may be represented in the cognitive disability group [17]. Our findings, therefore, may underrepresent the within-group diversity in sexual experiences, debut, and number of partners among people with disability. Our second limitation is related to the sensitive nature of sexual experience data. While the NSFG relies on computer aided self-interviewing, it is clear through the fluctuating analytic samples that some respondents chose not to answer some questions regarding sex. Another data limitation is that the NSFG only asks sexual abuse/coercion questions to those 18 years and older, focus only on heterosexual vaginal intercourse, and are restricted in the 2017–2019 waves. While our sensitivity analyses (Appendices B & C) show that voluntariness does not fully explain early sexual debut, we cannot be certain of consensualness of all sexual experiences across all ages, sex of partners, and data waves. Finally, given the cross-sectional nature of the data, cause and effect relationship between the study outcomes and independent variables of interest cannot be inferred.

Our study also has notable strengths. The NSFG provides a nationally representative sample of adolescent and young adult females. Further, by merging 2011–2019 waves, we obtained sufficient sample sizes to provide robust national estimates of sexual experiences among adolescent and young adult females with cognitive and physical/sensory disability. Stratifying the analyses showed that sexual experiences among disabled young females are not entirely homogenous. Further attention to the experiences of different disability groups is important in sex and sexuality research in order to craft more effective sex education strategies, responsive and accessible sexual and reproductive health care services, and address potential risks associated with a variety of sexual experiences. Moreover, research should consider why people with and without disabilities differ in their sexual experiences, debut, and number of partners.

## Supplementary Information

Below is the link to the electronic supplementary material.

Supplementary Material 1

Supplementary Material 2

Supplementary Material 3

## Data Availability

Raw data used in this article are publicly available from the National Center for Health Statistics at the Centers for Disease Control and Prevention. Data and associated documentation are stored by data wave years and can be found by navigating the NSFG website: https://www.cdc.gov/nchs/nsfg/index.htm.

## Footnotes

1 We use community-first and person-first language interchangeably throughout as per APA standards. Community-first language uses “disabled people” and person-first language uses “people with disability.”

2 We only have data on sex and not gender identity and have a mixed age group of females between ages 15 and 25. Therefore, we use “females” throughout the manuscript, in line with APA standards.
