# Supplementary material for: Inclusive Intimacy: Sexual Experiences, Debut, and Partners Among Females Ages 15–25 with and Without Disability, NSFG 2011–2019
Source: Sex Disabil. 2026 Feb 18;44(2):16. doi: 10.1007/s11195-025-09931-9 (PMC12916980; doi:10.1007/s11195-025-09931-9)
Supplement: Supplementary file 1 — Supplementary Material 1 [file 11195_2025_9931_MOESM1_ESM.docx]

Appendix A. Dependent Variable Missingness among Females Ages 15-25, NSFG 2011-2019 (n=7,884).

| Dependent Variable | Yes | No | Missing | Missing Reasons |
| --- | --- | --- | --- | --- |
| **Opposite-Sex Sexual Experiences** |  |  |  |  |
| Vaginal Intercourse | 5,237 | 2,647 | 0 |  |
| Give Oral Sex | 4,599 | 3,248 | 37 | 35 refused, 2 don't know |
| Receive Oral Sex | 4,771 | 3,076 | 37 | 32 refused, 5 don't know |
| Anal Sex | 1,726 | 6,126 | 32 | 28 refused, 4 don't know |
| 10 or more sexual partners | 824 | 4,840 | 2,220 | 2,131 "inapplicable*," 25 don't know, 63 refused |
| Sexual Debut <15 | 955 | 4,282 | 2,647 | 2,647 never had vaginal intercourse |
| **Same-Sex Sexual Experiences** |  |  |  |  |
| Any same-sex sexual experience | 1,477 | 6,394 | 13 | 13 "inapplicable" |
| Give Oral Sex | 894 | 6,977 | 13 | 12 refused, 1 don't know |
| Receive Oral Sex | 1,047 | 6,827 | 10 | 9 refused, 1 don't know |
| 10 or more sexual partners | 43 | 1,414 | 6,427 | 6,407 "inapplicable," 12 refused, 8 don't know |
| Sexual Debut <15 | 355 | 1,100 | 6,429 | 6,407 "inapplicable," 15 refused, 7 don't know |

*Inapplicable is the language provided by the National Survey of Family Growth
