# Supplementary material for: Inclusive Intimacy: Sexual Experiences, Debut, and Partners Among Females Ages 15–25 with and Without Disability, NSFG 2011–2019
Source: Sex Disabil. 2026 Feb 18;44(2):16. doi: 10.1007/s11195-025-09931-9 (PMC12916980; doi:10.1007/s11195-025-09931-9)
Supplement: Supplementary file 2 — Supplementary Material 2 [file 11195_2025_9931_MOESM2_ESM.docx]

Appendix B. Sensitivity Analysis for Voluntariness of First Vaginal Intercourse among Adult Females Aged 18-25, NSFG 2011-2017 (N=3,544).

|  | Total Sample (N=3,544) | | Voluntary (n=3,319) | | Involuntary (n=225) | |  |
| --- | --- | --- | --- | --- | --- | --- | --- |
|  | n | Weighted % | n | Weighted % | n | Weighted % | p-value |
| **Any Disability** |  |  |  |  |  |  | ^***^ p=0.000 |
| Not disabled | 2,768 | (79.1%) | 2,632 | (80.0%) | 136 | (63.2%) |  |
| Disabled | 776 | (20.9%) | 687 | (20.0%) | 89 | (36.8%) |  |
| **Cognitive Disability** |  |  |  |  |  |  | ^**^ p=0.004 |
| Not cognitively disabled | 3,006 | (85.0%) | 2,848 | (85.8%) | 158 | (72.8%) |  |
| Cognitively disabled | 538 | (15.0%) | 471 | (14.2%) | 67 | (27.2%) |  |
| **Physical/Sensory Disability** |  |  |  |  |  |  | ^**^  p=0.001 |
| Not phys/sens disabled | 3,226 | (91.6%) | 3,036 | (92.0%) | 190 | (83.5%) |  |
| Phys/sens disabled | 318 | (8.4%) | 283 | (8.0%) | 35 | (16.5%) |  |
| **Age Group** |  |  |  |  |  |  | p=0.090 |
| 18-21 years old | 1,509 | (43.9%) | 1,403 | (43.4%) | 106 | (52.0%) |  |
| 22-25 years old | 2,035 | (56.1%) | 1,916 | (56.6%) | 119 | (48.0%) |  |
| **Maternal Education** |  |  |  |  |  |  | p=0.238 |
| Less than high school | 733 | (17.9%) | 676 | (17.5%) | 57 | (25.0%) |  |
| High school graduate | 1,072 | (28.7%) | 1,006 | (28.7%) | 66 | (27.4%) |  |
| Some college | 975 | (27.7%) | 906 | (27.6%) | 69 | (28.2%) |  |
| College graduate | 764 | (25.8%) | 731 | (26.1%) | 33 | (19.4%) |  |
| **Family Poverty Status** |  |  |  |  |  |  | p=0.061 |
| 99% FPL or lower | 1,347 | (32.3%) | 1,237 | (31.8%) | 110 | (40.0%) |  |
| 100%-199% FPL | 860 | (23.1%) | 809 | (22.9%) | 51 | (27.1%) |  |
| 200%-399% FPL | 836 | (27.2%) | 787 | (27.3%) | 49 | (24.4%) |  |
| 400% FPL or higher | 501 | (17.5%) | 486 | (18.0%) | 15 | (8.5%) |  |
| **Race-Ethnicity** |  |  |  |  |  |  | p=0.134 |
| Non-Hispanic White | 1,563 | (53.4%) | 1,473 | (53.9%) | 90 | (44.1%) |  |
| Non-Hispanic Black | 806 | (16.3%) | 747 | (15.8%) | 59 | (24.1%) |  |
| Hispanic | 881 | (21.5%) | 828 | (21.5%) | 53 | (21.0%) |  |
| Non-Hispanic Other | 501 | (8.9%) | 271 | (8.8%) | 23 | (10.8%) |  |
| **Nativity** |  |  |  |  |  |  | p=0.396 |
| US Born | 3,194 | (90.3%) | 2,992 | (90.4%) | 202 | (87.3%) |  |
| Immigrant | 350 | (9.7%) | 327 | (9.6%) | 23 | (12.7%) |  |
| **Sexuality** |  |  |  |  |  |  | ^***^ p=0.000 |
| Straight | 3,110 | (89.2%) | 2,932 | (89.9%) | 178 | (77.2%) |  |
| Lesbian | 62 | (1.3%) | 53 | (1.2%) | 9 | (3.5%) |  |
| Bisexual | 372 | (9.5%) | 334 | (8.9%) | 38 | (19.3%) |  |
| **Data Wave** |  |  |  |  |  |  | p=0.678 |
| 2011-2013 | 1,286 | (33.9%) | 1,198 | (33.8%) | 88 | (36.2%) |  |
| 2013-2015 | 1,244 | (35.7%) | 1,163 | (35.6%) | 81 | (37.5%) |  |
| 2015-2017 | 1,014 | (30.4%) | 958 | (30.7%) | 56 | (26.4%) |  |

^*^p<0.05, ^**^p<0.01, ^***^p<0.00
