# Supplementary material for: Inclusive Intimacy: Sexual Experiences, Debut, and Partners Among Females Ages 15–25 with and Without Disability, NSFG 2011–2019
Source: Sex Disabil. 2026 Feb 18;44(2):16. doi: 10.1007/s11195-025-09931-9 (PMC12916980; doi:10.1007/s11195-025-09931-9)
Supplement: Supplementary file 3 — Supplementary Material 3 [file 11195_2025_9931_MOESM3_ESM.docx]

Appendix C. Sensitivity Analysis for Voluntary First Vaginal Intercourse, First Controlling for Voluntariness and Second Excluding Involuntary Intercourse Among Adult Females Aged 18-25, NSFG 2011-2017.

| Debut Variables | Any Disability (n=776 / 687) | | Cognitive Disability (n=538 / 471) | | Physical/Sensory Disability (n=318 / 283) | |  |
| --- | --- | --- | --- | --- | --- | --- | --- |
|  | Unadjusted PR (95% CI) | Adjusted PR (95% CI) | Unadjusted PR (95% CI) | Adjusted PR (95% CI) | Unadjusted PR (95% CI) | Adjusted PR (95% CI) | |
| Sexual Debut 14 and Under^1^ (n=3,544) | 1.86^***^ (1.47-2.35) | 1.57^***^ (1.25-1.97) | 1.69^***^ (1.29-2.20) | 1.41^*^ (1.08-1.84) | 2.15^***^ (1.60-2.89) | 1.89^***^ (1.42-2.53) | |
| Voluntary Sexual Debut 14 and Under^2^ (N=3,319) | 1.88^***^ (1.44-2.46) | 1.64^***^ (1.27-2.11) | 1.75^***^ (1.30-2.37) | 1.52^***^ (1.13-2.04) | 2.18^***^ (1.55-3.07) | 1.96^***^ (1.40-2.75) | |

^1^The first debut variable drops all missing data (Wave 4, Under 18 years old, Voluntariness, and Sexual Debut), and adjusts for voluntariness in the adjusted, modified Poisson regression models.

^2^The second debut variable drops the same missing data, but it also drops cases for whom their first vaginal sexual experience was involuntary.

Both sets of adjusted, modified Poisson regression models adjust for age group, poverty status, maternal education, race/ethnicity, nativity, sexuality, and data wave.
